# Mitochondrial proteins as therapeutic targets in diabetic ketoacidosis: evidence from Mendelian randomization analysis

**DOI:** 10.3389/fphar.2024.1448505

**Published:** 2024-10-14

**Authors:** Ruiqiang Xie, Hongyan Xie, Hong Gao, Chunguang Xie, Haipo Yuan, Zhijun Feng

**Affiliations:** ^1^ Hospital of Chengdu University of Traditional Chinese Medicine, Chengdu, Sichuan, China; ^2^ Jiangmen Central Hospital Postdoctoral Innovation Practice Base, Southern Medical University, Jiangmen, Guangdong, China

**Keywords:** mitochondrial proteins, diabetic ketoacidosis, mitochondrial dysfunction, Mendelian randomization, therapeutic targets, causal relationship

## Abstract

**Introduction:**

Diabetic ketoacidosis (DKA) is a severe and potentially fatal acute complication in diabetic patients, commonly occurring in type 1 diabetes (T1D) but also seen in type 2 diabetes (T2D). The pathogenesis of DKA involves complex physiological processes that are not fully understood, especially the role of mitochondria. Mitochondria, known as the powerhouse of cells, plays a crucial role in oxidative phosphorylation and ATP production, which is vital in various metabolic diseases, including diabetes. However, the exact causal relationship between mitochondrial dysfunction and DKA remains unclear.

**Methods:**

This study employed Mendelian randomization (MR) analysis and protein-protein interaction (PPI) networks to systematically explore the causal relationships between mitochondrial DNA copy number (mtDNA-CN) and specific mitochondrial proteins with DKA. We used bidirectional MR analysis and genome-wide association study (GWAS) data from openGWAS database to investigate the causal effects of mtDNA-CN and 64 mitochondrial-related proteins on DKA and its subtypes (T1DKA, T2DKA, unspecified-DKA).

**Results:**

The study revealed that increased mtDNA-CN significantly reduces the risk of DKA, whereas the effect of DKA on mtDNA-CN was not significant. Mitochondrial-related proteins such as MRPL32, MRPL33, COX5B, DNAJC19, and NDUFB8 showed a negative causal relationship with DKA, indicating their potential protective roles. Conversely, ATP5F1B and COX4I2 have a positive causal relationship with DKA, indicating that excessive ATP production in diabetic patients may be detrimental to health and increase the risk of severe complications such as DKA.

**Discussion:**

The results emphasize the necessity of protecting mitochondrial function in order to reduce the risk of DKA. The study offers novel perspectives on the molecular pathways involved in DKA, emphasizing the critical functions of mt-DNA and distinct proteins. These evidences not only enhance our comprehension of the implications of mitochondrial dysfunction in diabetes-related complications but also identify potential therapeutic targets for individualized treatment approaches, thereby making a substantial contribution to clinical care and public health initiatives.

## 1 Introduction

Diabetic ketoacidosis (DKA) represents a critical and potentially life-threatening acute complication observed in individuals diagnosed with diabetes (Pasquel et al., 2021). While it predominantly affects those with type 1 diabetes (T1D) (Rewers et al., 2021), it can also present in individuals with type 2 diabetes (T2D) (Karslioglu French et al., 2019). DKA is distinguished by elevated blood sugar levels, heightened ketone body production, and metabolic acidosis (Tran et al., 2017). Despite extensive efforts to elucidate the underlying mechanisms of DKA, certain intricate physiological processes, including mitochondrial involvement, remain poorly understood. Mitochondria are often described as cellular powerhouses, as they play a crucial role in energy metabolism processes like oxidative phosphorylation (OXPHOS) and ATP synthesis (Manevski et al., 2020; Shahandeh et al., 2021; Valach et al., 2023). Diabetes, a systemic metabolic disease, primarily manifests as long-term persistent hyperglycemia and metabolic disorders (Sarkar et al., 2018; Yang et al., 2022b). Thus, mitochondrial dysfunction and diabetes progression likely interact in complex ways. This relationship may lead to contributed decline in mitochondrial function, worsening metabolic balances and complicating treatments and recovery for diabetic patients. It may also increase the risk of complications like DKA.

Since molecular biology techniques have advanced, greater attention has been given to mitochondrial dysfunction in metabolic diseases (Elmore and La Merrill, 2019; Amorim et al., 2022; Park and Lee, 2022; Chen et al., 2023; Tang et al., 2024). Diabetes and its complications have been associated with mitochondrial DNA (mtDNA) mutations, mitochondrial biogenesis, and autophagy (Sharma, 2015; Zhang et al., 2021a; Hu et al., 2022; Deng et al., 2024). Researchers have found that diabetics are more likely to have mitochondrial genome instability than healthy individuals, suggesting mitochondrial genome instability may play a role in diabetes pathogenesis (Dabravolski et al., 2021). Mitochondrial biogenesis and autophagy are also considered markers of mitochondrial dysfunction, which is vital for maintaining mitochondrial population and cellular homeostasis (Wu et al., 2020; Munson et al., 2021). It has been observed in numerous studies that mitochondrial dysfunction is linked to diabetes and its complications (Yi et al., 2022; Gao et al., 2023; Lyssenko and Vaag, 2023; Yousef et al., 2023); however, there is n’t enough evidence to make a causal relationship. In order to better understand the role of mitochondria in DKA, prospective, randomized controlled studies are required. However, obtaining funding for such studies can be very challenging due to ethical and technical constraints. In light of this, Mendelian randomization (MR) analysis appears as a promising method for examining the causal relationship between mitochondrial dysfunction and DKA (Emdin et al., 2017).

MR analysis is a causal inference method that uses genetic variations as instrumental variables (IVs) (Davey Smith and Hemani, 2014). Genetic variations, established at birth and unaffected by environmental factors, underpin this method, inherently reducing confounding factors and enhancing the reliability of causal inference (Burgess et al., 2017b). This study employs MR analysis to explore the causal relationship between mitochondrial DNA copy number (mtDNA-CN), mitochondrial-related proteins, and DKA. The primary objectives are to establish causality through genetic variation evidence, provide new insights into DKA management with an emphasis on mitochondrial function, and support the development of mitochondrial-based interventions for prevention and treatment of DKA. By investigating these relationships, we aim to enhance the understanding and management of DKA in diabetic patients, potentially paving the way for novel preventive and therapeutic strategies.

## 2 Materials and methods

### 2.1 Study design and data source

By combining MR analysis and bioinformatics enrichment analysis, we explored potential regulatory mechanisms between mitochondria and DKA in this study. Firstly, based on genetic variation, we examined the bidirectional causal relationship between mtDNA-CN and DKA, considering that mtDNA encodes mitochondrial proteins. Secondly, we examined causal relationships between specific mitochondrial proteins and DKA and its subtypes (type 1 diabetes ketoacidosis, T1DKA; type 2 diabetes ketoacidosis; T2DKA, and unspecified diabetes ketoacidosis, UNDKA) based on genome-wide association study (GWAS) data on mitochondrial-related proteins. Thirdly, we developed a protein-protein interaction (PPI) network to identify key molecular modules and their associated biological processes, which deepened our understanding of mitochondria in DKA pathogenesis.

According to the STROBE-MR (Strengthening the Reporting of Observational Studies in Epidemiology using Mendelian Randomization) guidelines (Skrivankova et al., 2021), we conducted a bidirectional two-sample MR analysis. We obtained GWAS data on mtDNA-CN (Chong et al., 2022) (Supplementary Table S1, GWAS ID: ebi-a-GCST90026372, sample size: 383,476) and mitochondrial-related protein datasets (Sun et al., 2018) (a total of 64 proteins and 66 datasets are included, of which MUL1 and AIFM1 each contain two GWAS datasets) from the openGWAS database (https://gwas.mrcieu.ac.uk/, accessed on 10 May 2024). Additionally, DKA datasets were sourced from the FinnGen database (accessed on 10 May 2024) (Kurki et al., 2023). Detailed information regarding the datasets used in the current analysis and the genes encoding mitochondrial-related proteins can be found in Supplementary Table S1. These datasets include large sample sizes and are representative of ethnicities from different countries, effectively reducing sample overlap bias in MR analyses.

**FIGURE 1:**
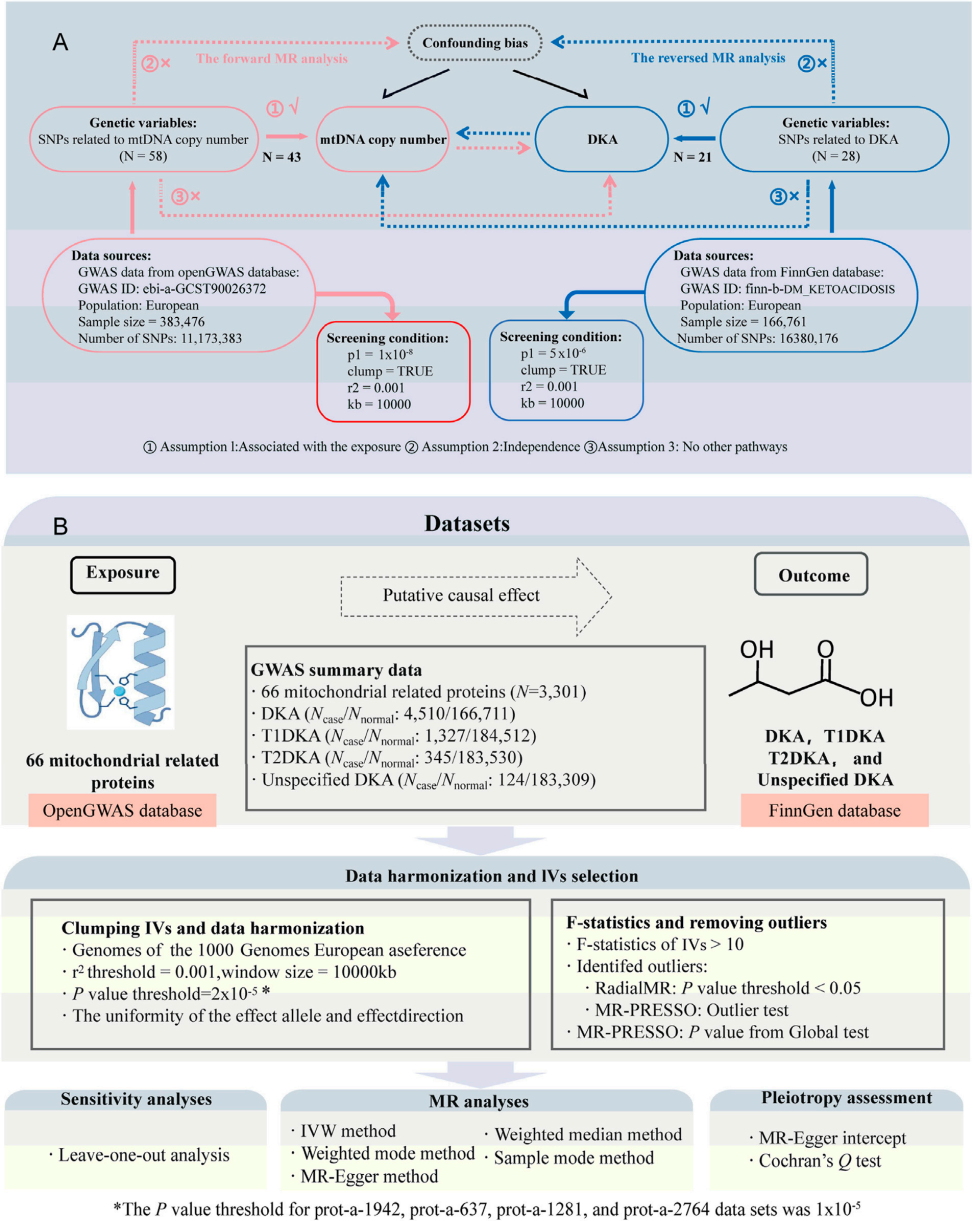
Study design and flowchart. **(A)**, flowchat for bidirectional Mendelian randomization (MR) analysis between mitochondrial DNA copy number (mtDNA-CN) and diabetic ketoacidosis (DKA); the part connected by the red line belongs to forward MR analysis; the part connected by the blue line belongs to inverse MR analysis; N, number of single nucleotide polymorphisms (SNPs). **(B)**, flowchart for MR analysis between mitochondrial-related proteins and DKA. *N*_case_, sample size of disease group; *N*_normal_, sample size of healthy group; T1DKA, type 1 diabetic ketoacidosis; T2DKA, type 2 diabetic ketoacidosis; IVW, inverse-variance weighted method.

During the first stage of the bidirectional MR analysis, mtDNA-CN was considered as the exposure and DKA as the outcome; in the reverse MR analysis, DKA as the exposure and mtDNA-CN as the outcome. In the second stage, mitochondrial-related proteins were analyzed as exposure, and DKA and its subtypes were analyzed as outcomes. Figure 1 illustrates the basic assumptions of the MR analysis, emphasizing the importance of IVs. Genetic variations related to exposure were selected as IVs, which must meet three key assumptions (Figure 1) (Lawlor et al., 2008): 1) IVs are strongly associated with exposure factors; 2) IVs are not related to confounders affecting both exposure and outcome; 3) IVs influence the outcome solely through the exposure. The data used in this study were derived from publicly available GWAS summary statistics, negating the need for additional ethical approval or informed consent.

Since mitochondrial function plays an important regulatory role and mitochondrial-related proteins interact with each other, we validated 64 unique mitochondrial mRNAs and constructed protein-protein interaction (PPI) network for them. Using the “MCODE” plugin in Cytoscape software (Shannon et al., 2003; Otasek et al., 2019), we identified key molecular modules. Based on the interactions within the key modules, we conducted in-depth analyses of biological processes and potential molecular regulatory pathways. These comprehensive analyses clarified the complex relationships between mitochondrial-related proteins and DKA and revealed their molecular interactions and pathway intersections. These findings provide new insights into the interaction mechanisms among these proteins and offer important information for developing potential therapeutic strategies and preventive measures.

### 2.2 Selection and quality control of IVs

During the IV quality control process, the following steps were followed to ensure validity and reliability: Firstly, for each exposure, nucleotide polymorphisms (SNPs) associated with that exposure were extracted, with different *p*-value thresholds applied. In the first stage (from mtDNA-CN to DKA analysis), *P* < 1 × 10^−8^ was used for the forward MR analysis but *P* < 5 × 10^−6^ was used for the reverse analysis. In the second stage (from mitochondrial-related proteins to DKA analysis), an initial threshold of *P* < 2 × 10^−5^ was set for SNP selection (Yan et al., 2024). However, subsequent analyses revealed horizontal pleiotropy in prot-a-1942, prot-a-637, prot-a-1281, and prot-a-2764 data set, respectively. In order to eliminate the influence of pleiotropy and ensure MR results that were reliable and stable, the SNP selection threshold was adjusted to *P* < 1 × 10^−5^ of these datasets. Linkage disequilibrium parameters *r* ^2^ = 0.001 and distance window kb = 10,000 were applied (Feng et al., 2024). *F*-statistics (*F* = *beta* ^2^/*se* ^2^) were calculated for each SNP, ensuring that all SNPs included in the analysis had *F*-statistics greater than 10 (Burgess et al., 2011; Liu et al., 2023). The LDlink database (https://ldlink.nih.gov/?tab=ldtrait) was used to screen for and exclude potential confounders (Lin et al., 2020), particularly IVs related to the outcome in different analysis directions. “RadialMR” (Bowden et al., 2018) and “MR-PRESSO” (Verbanck et al., 2018) methods were combined for IV quality control. “RadialMR” method and “Outlier test” in “MR-PRESSO” method were jointly used to identify potential outliers. Outliers identified in those two methods were excluded in the MR analysis. Finally, ensure that the IVs included in the analysis have a clear allele frequency (EAF). If this value is missing, we will supplement it with relevant data from the 1000 Genomes Project (Byrska-Bishop et al., 2022).

### 2.3 MR analysis and sensitivity analyses

The principal approach utilized for MR analysis in this investigation was the inverse-variance weighted (IVW) method (Burgess et al., 2013; Burgess et al., 2016), encompassing both forward and reverse analyses. Supplementary methods, such as MR Egger (Bowden et al., 2015), weighted median (Bowden et al., 2016), simple mode (Morrison et al., 2020), and weighted mode (Burgess et al., 2020), were also employed to comprehensively evaluate MR results. Robustness of the results was determined by their fulfillment of the following criteria simultaneously: 1) consistent direction of causal effects across all methods (i.e., all *B*-values > 0 for positive causal relationships or all *B*-values < 0 for reverse relationships); 2) attainment of a *p*-value below 0.05 using the IVW method (Harrison et al., 2018; Su et al., 2023). Sensitivity analyses included “MR-Egger intercept tests” to evaluate pleiotropy (*p* < 0.05 indicating pleiotropy) (Verbanck et al., 2018), “*Cochran’s Q test*” for heterogeneity (*p* < 0.05 indicating heterogeneity) (Bowden et al., 2017), and single SNP analysis to estimate causal effects. Leave-one-out analysis assessed the impact of individual SNPs on causal estimates (Burgess et al., 2017a). Scatter plots and funnel plots were used to display sensitivity analysis results. All statistical analyses were performed using R version 4.3.3, utilizing the “TwoSampleMR” (Hemani et al., 2017; Hemani et al., 2018), “MR-PRESSO” (Verbanck et al., 2018), and “RadialMR” packages (Bowden et al., 2018).

### 2.4 Mitochondrial related mRNA expression validation, PPI network construction and enrichment analysis

Utilizing the Gene Expression Omnibus (GEO) database (Clough and Barrett, 2016), we examined mRNA sequencing data from normal samples, classic T1D, and fulminant T1D (Nakata et al., 2013), specially analyzing the expression levels of 64 mitochondria-related mRNAs across these disease types. The risk of ketoacidosis is particularly high in patients with fulminant T1D. Thus, studying the gene expression patterns in these patients can provide a better understanding of the potential impact of changes in mitochondrial-related gene expression levels on their unique pathophysiological processes. The STRING database was used to create a PPI network for 64 mitochondrial-related proteins (Szklarczyk et al., 2023). The “MCODE” plugin (Otasek et al., 2019) in “Cytoscape” software was used to identify the key molecular modules in the interaction data. The STRING database provided quantitative evaluations of protein interaction strengths, and enrichment analyses identified related biological processes and molecular pathways. These results, quantified by “strength” data, aligned with the study’s objectives and helped interpret the findings. Detailed interaction networks were reconstructed in the STRING database for genes within key modules, and enrichment analysis results were obtained for Gene Ontology (GO), Kyoto Encyclopedia of Genes and Genomes (KEGG), and Reactome pathways. Corrected *p*-values < 0.05 were used to ensure statistical significance. Additionally, mitochondrial proteins identified as significant in MR analysis were highlighted in the PPI data. This approach not only enhanced understanding of the roles of mitochondrial-related proteins in DKA but also provided insights into how modulating their expression or activity could affect the pathological process with each other. These findings have the potential to identify molecular targets for new therapeutic strategies, offering fresh perspectives and methods for managing diabetes and its severe complications.

## 3 Results

### 3.1 Bidirectional causal relationship between mtDNA-CN and DKA

In the forward analysis of first stage, 58 SNPs closely related to mtDNA-CN were identified based on preset screening criteria. After excluding confounding IVs and performing quality control, 43 valid SNPs were included in the MR analysis (Figure 1; Supplementary Table S2). Similarly, in the reverse analysis of first stage, 28 SNPs closely associated with DKA were identified, and 21 valid SNPs were included in the MR analysis after subsequent exclusion and quality control (Figure 1; Supplementary Table S3). All 5 MR analysis methods in the forward analysis produced effect estimates less than 0, and the *p*-values from the weighted median and IVW methods were less than 0.05. Therefore, the forward analysis results support a negative causal relationship between mtDNA-CN and DKA (*B*_IVW_ = −0.58, *P*_IVW_ = 0.00055, Table 1). In contrast, no significant causal relationship was found in the reverse analysis (*P*_IVW_ = 0.98, Table 1). Additionally, sensitivity analyses of both forward and reverse analyses showed no significant heterogeneity (Table 1, *P*_*Q test*_>0.05) or pleiotropy (Table 1, *P*_egger_intercept_>0.05). The significance of the positive analysis results, combined with the lack of significant causal relationships in the reverse analysis, emphasizes the potential key role of mtDNA-CN in the onset and progression of DKA. This differential finding indicates that while increased mtDNA-CN may protect against DKA, the condition itself does not significantly influence mtDNA-CN. This asymmetry suggests that mtDNA-CN might be a proactive protective factor rather than a reactive element in response to DKA, highlighting its importance in cellular energy metabolism and physiological responses under diabetic stress conditions.

**TABLE 1:** The results of forward and inverse Mendelian randomization (MR) analyses.

Methods	*N*snp	*B*	*SE*	*P* value	Sensitivity analysis
Mitochondrial DNA copy number to Diabetic ketoacidosis					
MR Egger	43	−1.63E-02	3.60E-01	9.64E-01	*Heterogeneity*: *P*_*Q*__test_ = 0.941 *Pleiotropy*: *P*_*egger_intercept*_ = 0.083
Weighted median	43	−5.34E-01	2.48E-01	3.13E-02	
IVW	43	−5.82E-01	1.69E-01	5.50E-04	
Simple mode	43	−7.25E-01	4.53E-01	1.18E-01	
Weighted mode	43	−2.68E-01	3.05E-01	3.84E-01	
Diabetic ketoacidosis to mitochondrial DNA copy number					
MR Egger	21	−1.50E-04	2.04E-03	9.42E-01	*Heterogeneity*: *P*_*Q*__test_ = 0.967 *Pleiotropy*: *P*_*egger intercept*_ = 0.942
Weighted median	21	1.25E-03	1.98E-03	5.29E-01	
IVW	21	−4.32E-05	1.43E-03	9.76E-01	
Simple mode	21	1.04E-03	4.01E-03	7.98E-01	
Weighted mode	21	3.70E-04	1.53E-03	8.11E-01	

*N*snp, number of single nucleotide polymorphisms; *B*, estimated value of causal effects; *SE*, standard error; *Q* test, *Cochran’s Q* test to evaluate heterogeneity; *egger intercept*, the statistical parameter in MR-Egger regression to evaluate pleiotropy; IVW, inverse-variance weighted.

### 3.2 Sensitivity analysis of the first stage MR analysis

**FIGURE 2:**
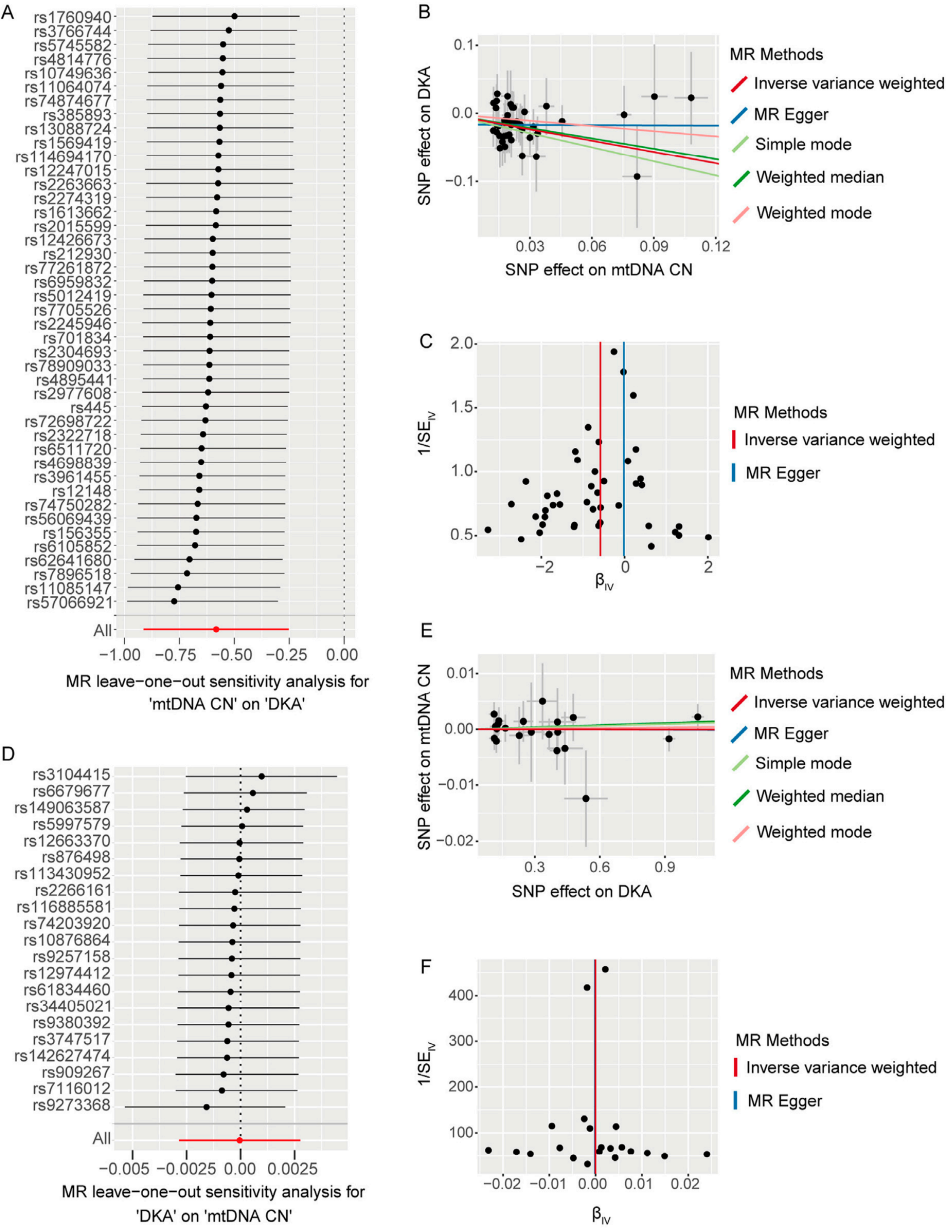
The results of leave-one-out analysis **(A, D)**, scatter plots **(B, E)**, and funnel plots **(C, F)** for bidirectional Mendelian randomization (MR) analysis between mitochondrial DNA copy number and diabetic ketoacidosis (DKA). **(A–C)** are for forward MR analysis, **(D–F)** are for inverse MR analysis. For forest plots **(A, D)**, the short horizontal black line corresponding to each single nucleotide polymorphism (SNP) represents the overall effect of the remaining SNPs on the outcome after removing the SNP in the current analysis. The dots represent the size of the estimated effect, and the width of the horizontal line represents the maximum value (right endpoint) and minimum value (left endpoint) of the effect. All, represents the overall causal effect in the current analysis. For scatter **(B, E)** and funnel plots **(C, F)**, each black dot represents the SNP included in the current analysis, and different colored lines represent the trend lines obtained by fitting causal effect estimates obtained under different methods (same to the legend).

We used the leave-one-out method to evaluate changes in the causal effect of remaining SNPs on the outcome after individually removing each SNP. In the forward analysis, the leave-one-out results indicated that the causal effect of the remaining SNPs on DKA remained negative and significant after removing any SNP included in the MR analysis (Figure 2A). The scatter plot in this direction (Figure 2B) showed a potential linear trend between the effects of the included SNPs on mtDNA-CN and DKA, while the funnel plot (Figure 2C) showed no abnormal distribution of the SNPs included in the MR analysis. In the reverse analysis, the leave-one-out results showed no significant causal effect of the remaining SNPs on mtDNA-CN after removing any SNP (Figure 2D). The scatter plot in this direction (Figure 2E) also did not show a significant linear trend, and the funnel plot (Figure 2F) similarly showed no abnormal distribution of the SNPs included in the MR analysis. These results further support a significant negative causal relationship between mtDNA-CN and DKA, while the causal effect of DKA on mtDNA-CN is not significant. These findings emphasize the potential key role of mitochondrial function in the pathogenesis of DKA and provide a solid foundation for future in-depth research on the potential mechanisms of mitochondria in diabetes and its complications. These insights have important implications for developing new clinical intervention strategies.

### 3.3 Causal effects of mitochondrial proteins on DKA

**FIGURE 3:**
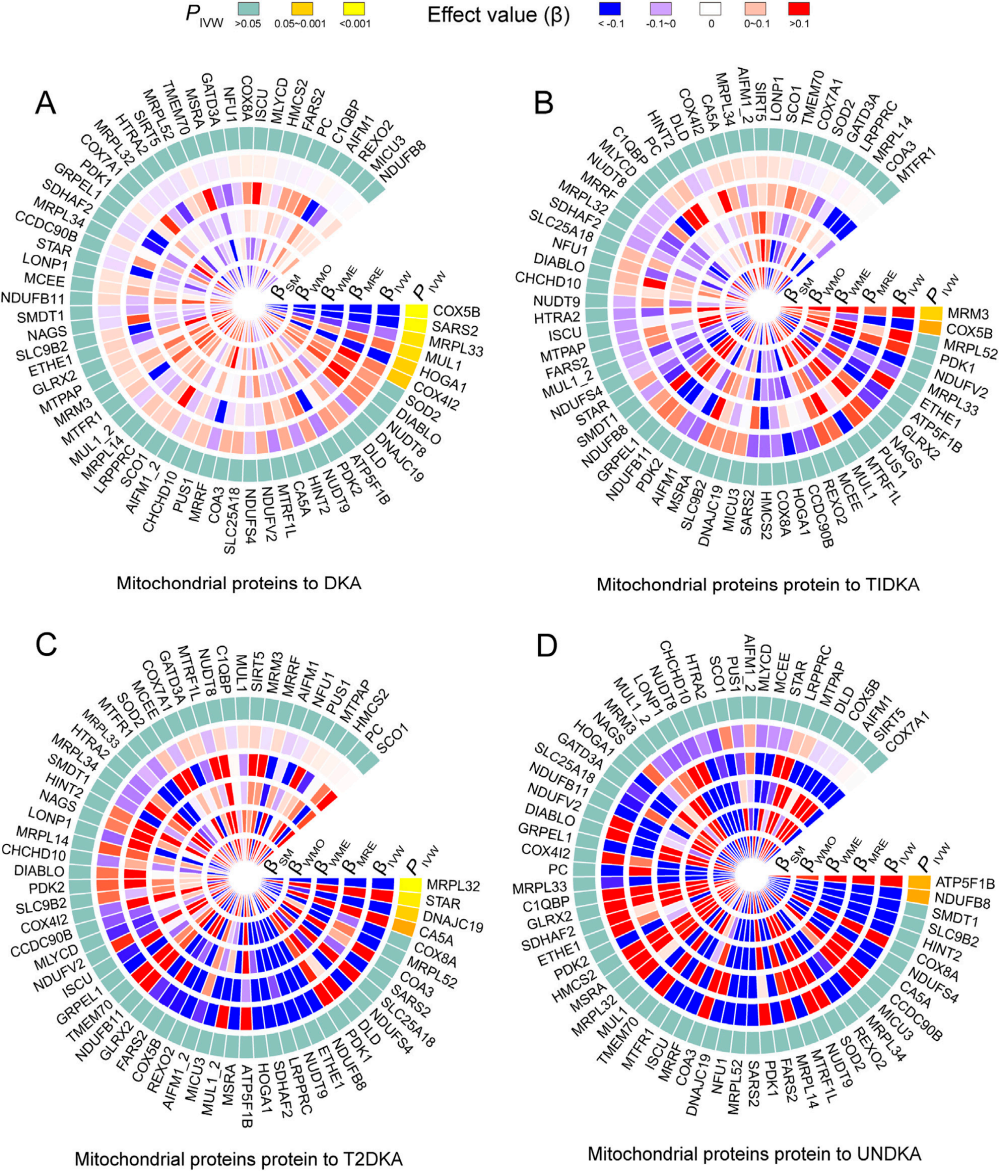
The heatmap shows the estimation of the causal effect of different types of mitochondrial related proteins on diabetes ketoacidosis **(A)** and its subgroups (type 1 diabetic ketoacidosis, **(B)** type 2 diabetic ketoacidosis, **(C)** unspecified diabetic ketoacidosis, **(D)** using Mendelian randomization (MR) analysis. The outermost circle of the heatmap represents the *p*-value obtained by the inverse-variance weighted (IVW) method. When the *p*-value is less than 0.05, indicating statistical significance, it is distinguished by different degrees of yellow. When the *p*-value is greater than 0.05, indicating no statistical significance, it is represented by light blue. The 1–5 layers in the heat map (from inside to outside) represent the estimated causal effects with simple mode (SM), weighted mode (WMO), weighted median (WME), MR-Egger (MRE) and inverse-variance weighted (IVW) methods. When the causal effect is positive (*β* > 0), it is represented by varying degrees of red; when the causal effect is negative (*β <* 0), it is represented by varying degrees of blue. DKA, diabetes ketoacidosis; T1DKA, type 1 diabetic ketoacidosis; T2DKA, type 2 diabetic ketoacidosis; UNDKA, unspecified diabetic ketoacidosis.

Based on the bidirectional MR analysis in the first stage, we confirmed a negative causal effect of mtDNA-CN on DKA. As mtDNA primarily encodes mitochondrial-related proteins, we further clarified the causal effect of mitochondrial-related proteins (66 GWAS datasets) on DKA by conducting MR analysis. The results showed that among the mitochondrial-related proteins with a causal effect on DKA (overall level), the following proteins exhibited a negative causal effect: cytochrome c oxidase subunit 5B (encoded by COX5B gene, Supplementary Table S1, GWAS ID: prot-a-638), serine-tRNA ligase (encoded by SARS2 gene, Supplementary Table S1, GWAS ID: prot-a-2627), 39S ribosomal protein L33 (encoded by MRPL33 gene, Supplementary Table S1, GWAS ID: prot-a-1942l), and 4-hydroxy-2-oxoglutarate aldolase (encoded by HOGA1 gene, Supplementary Table S1, GWAS ID: prot-a-1368) (Figure 3A, *P*_IVW_<0.05). In contrast, mitochondrial ubiquitin ligase activator of NFKB 1 (encoded by MUL1 gene, Supplementary Table S1, GWAS ID: prot-a-1970) and cytochrome c oxidase subunit 4 isoform 2 (encoded by COX4I2 gene, Supplementary Table S1, GWAS ID: prot-a-637) showed a positive causal effect on DKA (Figure 3A, *P*_IVW_ < 0.05).

In the subgroup analysis of DKA, it was found that rRNA methyltransferase 3 (encoded by MRM3 gene, Supplementary Table S1, GWAS ID: prot-a-2575) had a significant positive causal effect on T1DKA (Figure 3B, *P*_IVW_<0.05). Despite the statistical difference in the COX5B-encoding protein, inconsistent results in different methods indicated heterogeneity in T1DKA, so it was not considered significant. Additionally, 39S ribosomal protein L32 (encoded by MRPL32 gene, Supplementary Table S1, GWAS ID: prot-a-1941) and mitochondrial import inner membrane translocase subunit TIM14 (encoded by DNAJC19 gene, Supplementary Table S1, GWAS ID: prot-a-847) showed a negative causal effect on T2DKA (Figure 3C, *P*_IVW_ < 0.05), while steroidogenic acute regulatory protein (encoded by STAR gene, Supplementary Table S1, GWAS ID: prot-a-2866) showed a positive causal effect on T2DKA (Figure 3C, *P*_IVW_ < 0.05). Although carbonic anhydrase 5A (encoded by CA5A gene, Supplementary Table S1, GWAS ID: prot-a-332) showed statistical significance in the IVW method, the inconsistent effect trends across different methods prevented it from being considered a positive result in the current analysis. In the UNDKA subgroup, ATP synthase subunit beta (encoded by ATP5F1B gene, Supplementary Table S1, GWAS ID: prot-a-203) showed a positive causal effect on the outcome (Figure 3D, *P*_IVW_ < 0.05), while NADH dehydrogenase [ubiquinone] 1 beta subcomplex subunit 8 (encoded by NDUFB8 gene, Supplementary Table S1, GWAS ID: prot-a-2024) showed a negative causal effect on the outcome (Figure 3D, *P*_IVW_ < 0.05). All MR results in each analysis direction are provided in Supplementary Table S4. These findings suggest that certain mitochondrial-related proteins may play critical roles in the pathogenesis of DKA. These results not only reveal the complex interactions between mitochondrial-related proteins and DKA but also provide a solid foundation for further research on the role of mitochondria in diabetes and its complications. By further exploring the specific regulatory mechanisms of mitochondrial proteins in ketone body metabolism, we can better understand the pathogenesis of DKA and provide important references for developing new clinical intervention strategies.

### 3.4 Sensitivity analysis of the second stage MR analysis

**FIGURE 4:**
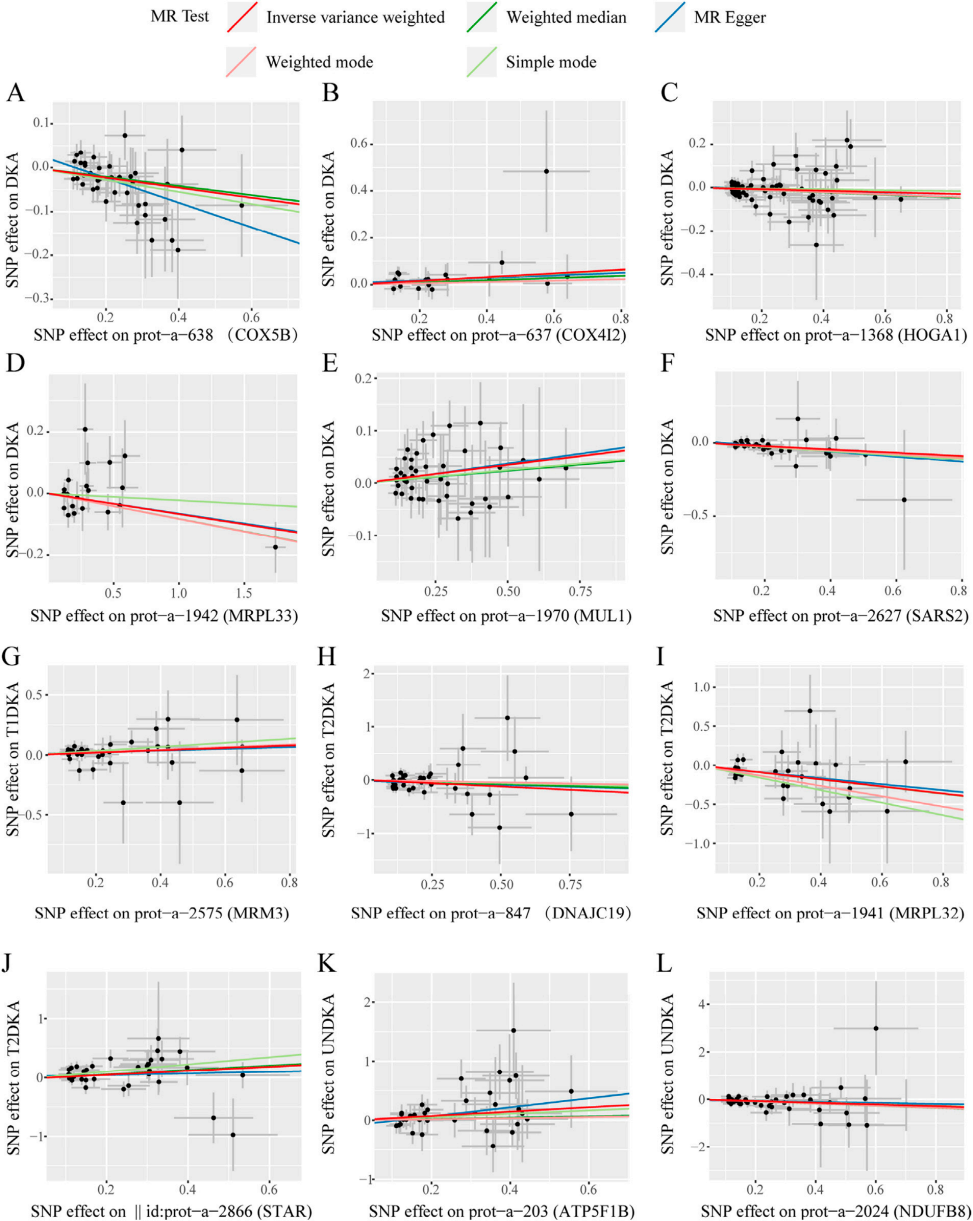
The scatter plots of statistically signifificant analysis directions in the MR analysis of mitochondrial-related proteins on diabetic ketoacidosis and its subgroups. **(A–L)** represent the directions of Mendelian randomization analyses for exposure (X-axis) and outcome (Y-axis). Each black dot represents the SNP included in the current analysis, and different colored lines represent the trend lines obtained by fitting causal effect estimates obtained under different methods (same to the legend). DKA, diabetes ketoacidosis; T1DKA, type 1 diabetic ketoacidosis; T2DKA, type 2 diabetic ketoacidosis; UNDKA, unspecified diabetic ketoacidosis.

In this stage, 12 mitochondrial-related proteins were found to have significantly different causal effects on DKA and its subgroups. The heterogeneity (Supplementary Table S5) and pleiotropy (Supplementary Table S6) assessments in each analysis direction did not show significant significance (*p* > 0.05). The scatter plots of the causal effects of the 12 mitochondrial-related proteins on DKA and its subgroups are shown in Figure 4. In these results, the causal effects of the exposure and the outcome estimated by the IVW method showed significant slope changes, and the distribution of SNPs did not show significant abnormal positions. The outcomes of the sensitivity analyses confirm the robustness of the current MR analysis, further solidifying the causal impact of mitochondrial-related proteins on the development of DKA, which underscores their potential as targets for therapeutic intervention to mitigate the risk and progression of DKA.

### 3.5 Mitochondrial gene expression varies notably across different diabetes types

**FIGURE 5:**
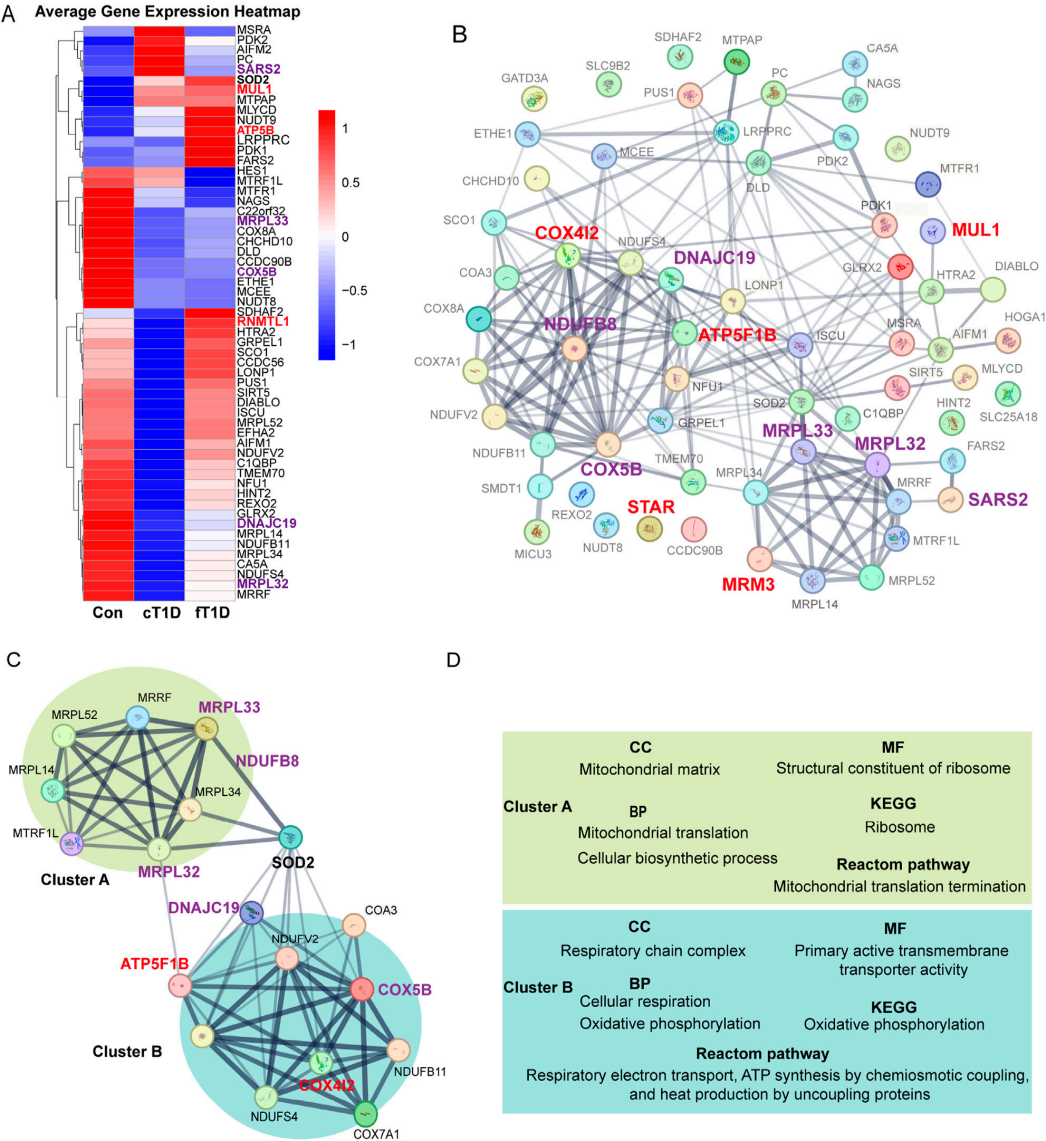
The expression differences for mitochondrial mRNA **(A)**, protein-protein interaction (PPI) networks between mitochondrial related proteins **(B)**, the key molecular modules **(C)**, and the results of enrichment analysis **(D)**. The enlarged and bold font in **(A–C)** represents mitochondria related proteins with statistical significance in Mendelian randomization (MR) analysis, where red font is used for positive causal effects between the proteins and diabetic ketoacidosis (including subgroups), and purple font is used for negative causal effects; the lines between two proteins represent the interaction between the two proteins, and the thickness of the lines represents the strength of the interaction (the thicker the lines, the stronger the evidence of interaction between the two proteins). **(C)**, a key molecular module obtained for the overall PPI **(B)**, where the interaction intensity of different genes shows two different clusters with enhanced interaction (cluster A and cluster B). **(D)**, the results of enrichment analysis for key genes in the key molecular model **(C)**, which mainly includes cellular components (CC), molecular functions (MF), biological processes (BP), Kyoto Encyclopedia of Genes and Genomes (KEGG) signaling pathway, and Reactome signaling pathway.

We validated the expression of 64 genes encoding mitochondrial proteins using the GSE44314 dataset. The results (Figure 5A) revealed differentiated expression patterns of these genes in various types of T1D patients. In classic T1D patients, genes represented by SARS2 exhibited significantly elevated expression levels. However, in fulminant T1D patients, SARS2 expression was notably downregulated, consistent with our current protein-level MR analysis indicating a negative causal relationship between SARS2 and DKA. Furthermore, we observed that the MUL1 gene was upregulated in both classic and fulminant T1D patients. Notably, ATP5B (also known as ATP5F1B) and RNMTL1 (also known as MRM3) genes showed significant upregulation primarily in fulminant T1D patients. Current MR analysis of these three proteins supported a positive causal effect on DKA. Conversely, COX5B, MRPL33, MRPL32, and DNAJC19 genes were significantly downregulated in fulminant T1D patients. MR analysis of these proteins supported a negative causal effect on DKA. These findings reveal differential expression patterns of mitochondria-related genes in various types of diabetes and their potential associations with DKA development, providing new insights into the molecular mechanisms of T1D, particularly fulminant T1D.

Using the STRING database, a PPI network encompassing 64 mitochondrial-related proteins was constructed. The results indicated that certain regions within the PPI network exhibited significantly enhanced interactions, and mitochondrial-related proteins with significant causal effects on DKA identified in current analyses were primarily concentrated in these regions (Figure 5B). After constructing key molecular modules, an 18-protein module was identified (Figure 5C). This module also contained two regions with significantly enhanced interactions: one represented by MRPL32 and MRPL33, named cluster A. Based on MR analysis results, the elevated expression levels of proteins in this cluster significantly reduced the risk of DKA (MRPL33 for DKA and MRPL32 for T2DKA), suggesting a protective role of mitochondrial proteins in cluster A in preventing DKA. The other region, represented by ATP5F1B, COX4I2, COX5B, DNAJC19, and NDUFB8, was named cluster B. According to MR analysis results, elevated expression levels of COX5B (for DKA), DNAJC19 (for T2DKA), and NDUFB8 (for UNDKA) in this cluster also significantly reduced the risk of DKA. However, elevated expression levels of ATP5F1B (for UNDKA) and COX4I2 (for DKA) increased the risk of DKA. This indicates that mitochondrial proteins in cluster B play a dual role in the pathogenesis of DKA, where some proteins may have protective effects, while others may exacerbate the condition. Furthermore, it is noteworthy that within this key molecular module, the primary link between Cluster A and Cluster B was established through Superoxide Dismutase 2 (SOD2) (Figure 5C). This suggests that SOD2 plays a critical role in coordinating mitochondrial protein function, particularly in managing oxidative stress and maintaining cellular homeostasis. SOD2’s bridging role highlights its importance in antioxidant defense and its potential function as a mediator between mitochondrial matrix functions and respiratory chain complex activities.

Based on evidence of interaction strengths, we analyzed the primary biological processes and potential molecular signaling pathways within key molecular modules. In terms of molecular composition, cluster A primarily includes mitochondrial matrix components, while cluster B mainly comprises respiratory chain complex components (Figure 5D; Supplementary Table S7). In terms of molecular function, cluster A is primarily associated with ribosomal structural components, whereas cluster B is related to the regulation of transmembrane transporter activity (Figure 5D; Supplementary Table S7). In biological processes, cluster A is mainly involved in mitochondrial migration and cellular biosynthesis processes, whereas cluster B is related to cellular respiration and oxidative phosphorylation (Figure 5D; Supplementary Table S7). KEGG pathway analysis results indicate that cluster A primarily affects ribosomal function, while cluster B is involved in regulating oxidative phosphorylation (Figure 5D; Supplementary Table S7). Reactome pathway analysis results show that cluster A mainly impacts mitochondrial translation processes, while cluster B is involved in respiratory electron transport, chemiosmotic coupling of ATP synthesis, and uncoupling protein-mediated thermogenesis (Figure 5D; Supplementary Table S7). Overall, the function of the mitochondrial matrix helps reduce the risk of DKA, while the oxidative phosphorylation process and the functional state of the respiratory chain complex have dual roles in the occurrence and development of DKA. These findings indicate that specific mitochondrial proteins may play critical roles in the pathogenesis of various DKA. The different biological processes and molecular functions represented by cluster A and cluster B reveal the complex mechanisms by which mitochondria participate in cellular energy metabolism and maintain cellular functions. They also reflect the multifaceted roles of mitochondrial-related proteins in the progression of DKA.

## 4 Discussion

This study employed MR and integrated bioinformatics analysis to systematically explore the causal relationships between mtDNA-CN, mitochondrial-related proteins and DKA. The investigation began with a bidirectional MR analysis, revealing a significant negative causal effect of mtDNA-CN on DKA, whereas no significant causal relationship was observed from DKA to mtDNA-CN. Given that mitochondrial DNA copy number encodes major mitochondrial proteins, the evidence of a negative causal relationship between mitochondrial DNA copy number and DKA also suggests potential effects of mitochondrial proteins on DKA. Based on this hypothesis, we subsequently utilized genome-wide association study (GWAS) data for 64 mitochondrial proteins to further analyze the causal relationships between these proteins and DKA, as well as its disease subtypes. Meanwhile, we employed bioinformatics approaches to thoroughly investigate the interaction networks among mitochondrial proteins and identify key regulatory proteins, aiming to comprehensively elucidate the potential role of mitochondrial proteins in the pathogenesis of DKA. Results shown that proteins encoded by MRPL32 and MRPL33 genes, which affect mitochondrial matrix, migration, and mitochondrial ribosomal functions, are associated with a reduced risk of DKA (MRPL33 for DKA and MRPL32 for T2DKA). Conversely, the proteins, encoding by ATP5F1B, COX4I2, COX5B, DNAJC19, and NDUFB8 genes, which relate to mitochondrial respiratory chain functions and OXPHOS processes, and exhibited complex dual effects on DKA. Specifically, increased expressions of COX5B (for DKA), DNAJC19 (for T2DKA), and NDUFB8 (for UNDKA) contribute to a decreased risk of DKA, while increases in ATP5F1B (for UNDKA) and COX4I2 (for DKA) expressions may enhance the DKA risk. Additionally, we established a positive causal relationship between the protein encoded by MRM3 gene and T1DKA. Through comprehensive application of bioinformatics analysis methods, we verified the differential expression levels of mitochondria-related genes in diabetic patients at the mRNA level. Additionally, we conducted PPI network analysis, clustering analysis, and functional enrichment analysis on mitochondria-related proteins. The results indicate that the integrity of fundamental mitochondrial functions (such as biosynthesis, migration, and protein translation) and mitochondrial structure are critical factors influencing the onset and progression of DKA. These findings provide valuable evidence for understanding the characteristic changes in mitochondrial metabolism during the development and progression of DKA from a molecular mechanistic perspective.

Based on this evidence, we propose several potential pharmacological intervention strategies. Firstly, we advocate for the development of drugs aimed at optimizing mitochondrial energy metabolism. Specifically, targeting MRPL32 and MRPL33, we suggest the design of small molecule compounds that selectively bind to and modulate their activity. These compounds could potentially intervene in mitochondrial protein synthesis by regulating the assembly or function of mitochondrial ribosomes. Additionally, for COX5B, we recommend the development of compounds capable of influencing the activity of cytochrome C oxidase, thereby modulating the function of the mitochondrial respiratory chain. Secondly, regulating cellular stress response is also an important direction. As an example, we can design peptide drugs that mimic the functional domains for DNAJC19 protein. These peptides can enhance cellular resistance to metabolic stress by inhibiting DNAJC19 activity through competitive binding. Furthermore, RNA interference technology and antisense oligonucleotides can be used to precisely control the expression levels of target proteins, especially when proteins such as ATP5F1B and COX4I2 have to be inhibited. It is our hope that these directions and strategies will be systematically explored and developed in order to translate the fundamental findings of this study into practical clinical interventions.

Subgroup analysis indicates that different mitochondrial proteins have unique causal impacts on various types of DKA, notably with the risk of T1DKA significantly influenced by an increase in MRM3 expression. From the perspective of molecular function, MRM3 is involved in the methylation of mitochondrial rRNA, a crucial step that ensures proper assembly and function of mitochondrial ribosomes (Cheng et al., 2021; Rebelo-Guiomar et al., 2022). Consequently, increased expression of MRM3 may enhance the efficiency of mitochondrial protein synthesis, maintain mitochondrial structural stability, and improve the efficiency of oxidative phosphorylation and antioxidant stress capacity (Rorbach et al., 2014). However, in diabetic patients, overexpression of MRM3 could further exacerbate metabolic imbalances, leading to the accumulation of metabolic products and thereby increasing the risk of DKA. Furthermore, excessive expression of MRM3 might disrupt mitochondrial quality control mechanisms, such as autophagy and protein degradation, potentially harming mitochondrial health and function and increasing the risk of DKA. Based on the molecular functions of mitochondrial proteins with significant MR analysis, we found that T2DKA is primarily associated with imbalances in both mitochondrial function and capacity, while UNDKA is mainly related to imbalances in mitochondrial capacity. These findings provide crucial guidance for the development of mitochondrial-targeted therapeutic strategies for DKA. Firstly, ensuring the integrity of mitochondrial structure and function is vital, particularly in maintaining the mitochondrial matrix and ribosomal functions, and in synthesizing mitochondrial proteins. Secondly, attention should be given to proteins related to OXPHOS and respiratory chain functions, with a dynamic assessment of mitochondrial capacity balance. Overall, this study not only deepens our understanding of the pathophysiological mechanisms of DKA from a mitochondrial perspective but also offers significant theoretical support for the development of new treatment strategies.

MR analysis, using genetic variants as IVs, aims to assess the causal relationships between exposures and outcomes (Davey Smith and Hemani, 2014). In this study, MR analysis confirmed a significant negative causal effect of mtDNA-CN on reducing the risk of DKA. Additionally, various mitochondrial-related proteins exhibited differing causal effects on DKA. Overall, through the study of genetic variations, we obtained direct evidence of a link between mitochondrial function and DKA risk, providing a theoretical basis for further investigating the interactions between the two. From a clinical perspective, these findings emphasize the importance of assessing mitochondrial function integrity in diabetes management. Diabetes patients are highly susceptible to mitochondrial dysfunction (Phielix et al., 2008; Chattopadhyay et al., 2011; Pinti et al., 2019), as evidenced by this study and published literature. Therefore, an evaluation system for mitochondrial function for diabetics is essential. This system should first monitor the integrity of mitochondrial structure and function, including ribosomal functions, to ensure efficient energy production processes; secondly, it should focus on mitochondrial OXPHOS levels, as this relates to the activity of the mitochondrial electron transport chain, directly impacting cellular energy metabolism. Additionally, this evaluation system can not only help clinicians better understand patients’ metabolic states but also provide more targeted treatment recommendations, thus optimizing the treatment of diabetes and its complications.

mtDNA-CN, the number of mtDNA copies per cell (Morin et al., 2022), is crucial for mitochondria to generate sufficient energy to maintain cellular functions (Malik and Czajka, 2013; Ashar et al., 2017). In the diabetic state, especially when complicated by DKA, the demand for energy in cells significantly increases, making mitochondrial efficiency particularly important. Physiologically, an increase in mtDNA copy number typically also leads to an increase in the synthesis of mitochondrial respiratory chain proteins, aiding in the efficiency of mitochondrial OXPHOS. Additionally, when the mtDNA-CN is sufficiently high, mitochondria can effectively meet the increased energy demands of the body in a short time, reducing the cells’ reliance on anaerobic glycolysis, thus lowering the accumulation of lactate and ketone bodies (Chen et al., 2022; Chong et al., 2022), which is crucial in preventing the onset and progression of DKA. Moreover, existing reports confirm that oxidative stress plays a central role in the pathogenesis of DKA (Rains and Jain, 2011; Li and Shen, 2018; Yurista et al., 2021). Higher levels of mtDNA-CN mean that mitochondria have sufficient capacity to participate in the clearance of ROS, thereby reducing the levels of oxidative stress in the body (Wang et al., 2019; Vakrou et al., 2021; Shi et al., 2022), which primarily mediated by the mitochondrial antioxidant system, consisting mainly of superoxide dismutase (SOD) and glutathione peroxidase (Vehviläinen et al., 2014; Kitada et al., 2020; Zhang et al., 2021b; Bao et al., 2022). In our analysis, we found that SOD2 occupies a central position in the key molecular module connecting mitochondrial ribosomal functions and the oxidative respiratory chain. This finding highlights the key role of SOD2 in maintaining mitochondrial homeostasis. According to the results from MR analysis, we have established a negative causal relationship between mtDNA-CN and DKA. However, reverse MR analysis did not reveal a significant causal effect of DKA on mtDNA-CN. Considering the role of mtDNA-CN in maintaining mitochondrial structural integrity and functional normalcy, we can summarize these findings as follows: Firstly, the dynamic balance of mtDNA-CN helps maintain mitochondrial function, thereby reducing endoplasmic reticulum stress and improving cellular adaptation to the diabetic environment (Sutton-McDowall et al., 2016; Sadakierska-Chudy et al., 2017; Hirano et al., 2021). Secondly, an appropriate increase in mtDNA-CN can also help improve mitochondrial metabolic adaptability, support more fatty acid oxidation, and potentially reduce excessive ketone production by enhancing the effective utilization of fatty acids (Watson et al., 2019; Xu et al., 2019; Venit et al., 2023). According to these evidences, mtDNA CN levels can reduce the risk of DKA significantly and explain the negative causal association between mtDNA CN and DKA. However, given that DKA is an acute and severe condition, the body undergoes intense metabolic disturbances and energy imbalances. In this context, we can explain why DKA does not exert a significant causal effect on mitochondrial DNA copy number (mtDNA-CN) from several perspectives: First of all, mitochondrial DNA replication and degradation are relatively slow processes, and DKA does not adversely affect mitochondrial DNA CN in the short-term. Secondly, in the DKA state, the body prioritizes various metabolic emergency mechanisms to maintain vital signs stability, such as increasing gluconeogenesis, lipolysis, and ketone body production. These acute compensatory mechanisms may temporarily mask potential effects on mtDNA-CN. Thirdly, although DKA leads to severe metabolic disturbances, the body still strives to maintain basic mitochondrial functions to support cellular energy supply. This compensatory mechanism may, to some extent, protect mitochondrial DNA stability, thus weakening the direct impact of DKA on mtDNA-CN. Overall, current MR results emphasize the potential upstream regulatory role of mtDNA-CN in the pathogenesis of DKA, rather than being a consequence of the disease. This key evidence reveals that mtDNA-CN significantly influences the biological processes of DKA through multiple pathways, including regulation of mitochondrial energy metabolism efficiency, enhancement of cellular antioxidant capacity, modulation of cellular stress response mechanisms, and improvement of metabolic adaptability.

Our analysis of mitochondria-related mRNA expression levels revealed that both classical T1D and fulminant T1D patients exhibit significantly different mRNA expression patterns compared to healthy individuals. Notably, fulminant T1D patients demonstrated more pronounced changes in the expression of mitochondrial function-related genes. These changes include downregulation of genes associated with mitochondrial DNA replication, transcription, and translation, as well as upregulation of genes related to mitochondrial energy metabolism and oxidative stress response. This evidence emphasizes the potentially crucial role of mitochondrial dysfunction in the pathogenesis of T1D, particularly in fulminant T1D, providing new insights into understanding the rapid progression and severity of the disease. Additionally, a key molecular module was identified through a PPI network among mitochondrial-related proteins. This module includes areas of increased interaction strength represented by the MRPL family proteins and another area represented by ATP5F1B, COX4I2, COX5B, DNAJC19, and NDUFB8. Notably, these two different genomic compositions of enhanced interaction areas are connected by the key antioxidant enzyme superoxide dismutase 2 (SOD2), showing potential functional synergy. In the MR analysis, proteins with causal effects on DKA were also mainly concentrated in this key molecular module. These findings emphasize the core role of mitochondrial proteins in maintaining cellular energy balance and responding to oxidative stress, especially in the pathophysiological process of DKA. This provides an important theoretical basis for studying the interaction between mitochondrial dysfunction and diabetic ketoacidosis and may have implications for developing new treatment strategies targeting DKA.

Mitochondrial ribosomal proteins, such as MRPL32 and MRPL33, are key components of the large subunit of the mitochondrial ribosome (Houtkooper et al., 2013; Kim et al., 2023). These proteins are primarily responsible for protein synthesis within mitochondria, and the proteins synthesized constitute the mitochondrial electron transport chain and other critical metabolic pathways, thus forming the basis for mitochondrial oxidative phosphorylation capacity (Wang et al., 2021). MRPL32 and MRPL33, by participating in this protein synthesis, directly enhance the biosynthetic capacity and energy production efficiency of mitochondria (Yang et al., 2022a). If mitochondrial ribosomal protein function is impaired, it could lead to reduced efficiency of mitochondrial protein synthesis, thereby affecting the overall function of mitochondria, impacting cellular energy balance and survival capacity (Lax et al., 2015). In this study, we confirmed that increased expression levels of MRPL32 and MRPL33 are associated with a reduced risk of DKA, likely due to these proteins enhancing mitochondrial metabolic efficiency and energy production capacity, allowing cells to more effectively cope with metabolic stress under hyperglycemic conditions. This finding emphasizes the importance of maintaining mitochondrial protein synthesis capacity to prevent metabolic disorders.

In the mitochondrial electron transport chain, COX5B is a member of complex IV, which is critical for electron transfer and proton pump activity, which is directly linked to ATP synthesis (Soro-Arnaiz et al., 2016; Gottschalk et al., 2022). Thus, the function of COX5B directly impacts the energy output efficiency of mitochondria. DNAJC19 (DnaJ Heat Shock Protein Family (Hsp40) Member C19) is a DnaJ protein located on the mitochondrial inner membrane, involved in protein folding and protein transport on the mitochondrial membrane. The function of DNAJC19 is essential for maintaining the integrity and function of the mitochondrial inner membrane, and it is also very important for the correct assembly and maintenance of protein complexes in the mitochondrial electron transport chain (Richter-Dennerlein et al., 2014; Wachoski-Dark et al., 2022). NDUFB8 (NADH Dehydrogenase [Ubiquinone] 1 Beta Subcomplex Subunit 8) is part of complex I of the mitochondrial electron transport chain, responsible for the oxidation of NADH and the transfer of electrons to ubiquinone. Thus, NDUFB8 plays a core role in maintaining the overall function and efficiency of the electron transport chain (Wu et al., 2016; Sun et al., 2017; Piekutowska-Abramczuk et al., 2018). The negative causal relationships between COX5B, DNAJC19, NDUFB8, and DKA indicate that enhanced functions of these proteins may reduce the risk of DKA by reducing oxidative stress and improving the efficiency of the mitochondrial respiratory chain.

In the present analysis, the positive causal relationships between the terminal components of the mitochondrial respiratory chain, specifically COX4I2 and ATP5F1B, and DKA, may reflect metabolic imbalances caused by the overactivation of these proteins under conditions of high energy demand and stress. Particularly in the diabetic milieu, hyperglycemia compels cells to rely more heavily on mitochondrial oxidative phosphorylation for energy production, thereby increasing the demand for ATP synthesis. COX4I2, a part of cytochrome c oxidase, is responsible for maintaining the activity of complex IV, especially under hypoxic conditions (Sommer et al., 2017). Its regulatory role ensures the efficiency of the OXPHOS process, supporting effective electron transfer and energy production (Li et al., 2022; Mao et al., 2022; Zhang et al., 2023). However, this efficient energy generation might lead to further disruption of the cellular environment in the context of DKA, such as by increasing the production of reactive oxygen species (ROS) or exacerbating intracellular acidosis, thus indirectly promoting the progression of DKA. ATP5F1B, part of complex V, also known as ATP synthase or complex F (Ganetzky et al., 2022; Sharma et al., 2023), plays a crucial role in cellular energy metabolism by synthesizing ATP from ADP and inorganic phosphate through a chemiosmotic coupling process (Nguyen et al., 2021; Fomo et al., 2022; Slater et al., 2022). Enhanced activity of ATP5F1B might lead to excessive ATP production, beneficial under normal circumstances but potentially exacerbating metabolic disturbances during DKA. Therefore, while the enhanced functions of ATP5F1B and COX4I2 support cellular metabolism under normal conditions, in the pathological state of diabetes and especially DKA, they might intensify metabolic stress and cellular stress responses. This highlights the need to consider the complexity and dual nature of mitochondrial function regulation when designing treatment strategies for diabetic patients, particularly in managing DKA or other severe metabolic disturbances.

Based on our current analysis results, published literature evidence, and the physiological functions of mitochondrial proteins, we summarize the mechanisms by which mitochondria participate in the occurrence and development of DKA as follows: Firstly, higher levels of mtDNA-CN can significantly reduce the risk of DKA through multiple pathways, including enhancing mitochondrial energy metabolism efficiency, improving cellular antioxidant capacity, regulating cellular stress response mechanisms, and enhancing metabolic adaptability. Secondly, mitochondrial protein synthesis and structural integrity are crucial for preventing DKA. Thirdly, mitochondrial respiratory chain function and oxidative phosphorylation (OXPHOS) processes play a complex dual role in the occurrence and development of DKA. On one hand, enhanced function of certain respiratory chain components (such as COX5B, DNAJC19, and NDUFB8) may reduce DKA risk by decreasing oxidative stress and improving mitochondrial respiratory chain efficiency. On the other hand, overactivation of terminal respiratory chain components (such as COX4I2 and ATP5F1B) may exacerbate metabolic imbalances and cellular stress responses under diabetic and DKA pathological conditions, potentially increasing DKA risk. Fourth, the mitochondrial antioxidant system, especially superoxide dismutase (SOD2), plays a central role in maintaining mitochondrial homeostasis and connecting different functional modules. Overall, these findings not only deepen our understanding of the pathophysiological mechanisms of DKA but also provide important theoretical support for developing new DKA prevention and treatment strategies, particularly in assessing and maintaining mitochondrial functional integrity, balancing mitochondrial capacity, and targeting mitochondrial function regulation.

Despite this study’s innovative use of MR analysis and mitochondrial protein interaction networks to reveal the causal relationships between mtDNA-CN and specific mitochondrial proteins such as MRPL32, MRPL33, COX5B, DNAJC19, and NDUFB8 with DKA, and a deep analysis of their molecular mechanisms, there are limitations. Firstly, while MR analysis provides insights into causal relationships, it relies on genetic variants as instrumental variables. This approach may not capture all aspects of complex biological processes involved in DKA development. Secondly, the study may be limited by the sample size and population characteristics of the GWAS data used for mitochondrial proteins. The generalizability of findings to diverse populations needs further validation. Thirdly, the study provides a snapshot of genetic associations but may not fully capture the dynamic nature of mitochondrial function and DKA development over time. Future research may be able to validate and extend these findings, based on these limitations.

## 5 Conclusion

This study, through combined analysis of MR and integrated bioinformatics, reveals a causal relationship between mitochondrial function and DKA risk. Increased mtDNA-CN and expression of specific mitochondrial proteins (MRPL32, MRPL33, COX5B, DNAJC19, NDUFB8) are associated with reduced DKA risk, while elevated ATP5F1B and COX4I2 levels correlate with increased risk. These findings underscore the importance of mitochondrial integrity in DKA pathogenesis and provide a foundation for developing mitochondrial-targeted therapies. Our results suggest that modulating mitochondrial protein expression and maintaining mitochondrial function could be effective strategies for DKA prevention and management.

## Data Availability

The publicly available datasets that were generated and/or analysed during the current study are available in the ieu open GWAS project (https://gwas.mrcieu.ac.uk/).
